# Bioethics in biomedicine in the context of a global higher education area

**DOI:** 10.1186/1755-7682-3-10

**Published:** 2010-06-11

**Authors:** Antonio Liras, Alicia Arenas

**Affiliations:** 1 Department of Physiology, School of Biological Sciences, Complutense University of Madrid, Spain

## Abstract

The University is tasked with drawing together, transmitting and maintaining knowledge, while creating an area where the ethical "sense" required for working in the field of Biology and Biomedicine can be provided. Although scientific knowledge is present on an overwhelming scale in nature and, therefore, its discovery is unceasing, this does not mean that, as a human being, the researcher has no limitations. It is Bioethics that sets this limit. The successful spreading of knowledge, therefore, which is proclaimed with the creation of a Global Higher Education Area, should also pursue the establishment of the bioethical principles necessary for the credibility of science and its progress so that the society that it promotes and sustains becomes a reality.

## International and Global Bioethics: State of the Art

In 1998, Baker [1] proffered an alternative rationale for international bioethics based on the fact that international bioethics can be reconstructed as a negotiated moral order that respects culturally and individually defined areas. The theory of a negotiated moral order is flexible to absorb the genuine insights of multiculturalism. This theory is consistent with several controversies such as the controversy over changing the consent rule for experiments in medicine and the controversy over exempting certain clinical trials.

Individual human rights in the field of health care have been implemented by most international organisations, including the European Union and the World Health Organisation. The Council of Europe is, however, particularly prominent in its work in the field of human rights, thanks to the Convention on Human Rights and Biomedicine, which strengthens on an international level the legal position of the patient and the research subject in setting a minimum level of protection in respect of individual human rights and health [2]. Bioethics and human rights are two different systems of norms and bioethics can enrich human rights by extending the traditional catalogue of rights in new fields [3].

The Council of Europe Convention on Human Rights and Biomedicine is the first legally binding international biomedical law and ethics document to uphold human dignity and to provide a legal framework for societies with different sociocultural and philosophical backgrounds [4]. Human dignity (moral sense), is a term used in moral, ethical, and political discussions to signify that a being has an innate right to respect and ethical treatment. It is an extension of Enlightenment-era beliefs that individuals have inherent, inviolable rights, and thus is closely related to concepts like virtue, respect, self-respect, autonomy, human rights, and enlightened reason. The most prominent references to dignity appear in the many international human rights instruments, such as the United Nations' universal declaration of human rights, and with few exceptions, these conventions do not address medical treatment or research. A leading exception is the Council of Europe's convention for the protection of human rights and dignity of the human being with regard to the application of biology and medicine [5,6]. At the other pole, Macklin proposes that the dignity is a useless concept in medical ethics and it can be eliminated without any loss of content [7].

The principle of respect for human dignity plays a crucial role in the emerging global norms relating to bioethics, in particular in the UNESCO Universal Declaration on Bioethics and Human Rights [8,9]. UNESCO is an intergovernmental organization with 193 Member States. It is concerned with a broad range of issues regarding education, science and culture. Since 1993 it has been addressing the ethics of science and technology, with special emphasis on bioethics. Its major objective being the development of international regulatory standards, particularly for Member States with a limited infrastructure in bioethics and educational programs, lacking expertise in bioethics committees and legal frameworks.

The main question is the relationship between human rights and ethics, and what are the advantages of norms of bioethics over international human rights in relation to a public health issues or to scientific research. Bioethics is certainly a decentralized normative system and has a traditional appeal to many of the health professionals involved [10,11]. There are at least three aspects, in biosciences, related to ethics and human rights [12]: i) Goals of the individual with respect to level of health and quality of life; ii) Social action and reform to increase the availability of care and to facilitate access to needed health care for all, and iii) Patient education and advocacy to ensure that individuals are aware of all options about health care. As Robinson say [13], ethics, human rights and globalization are part of our everyday experience and their interactions with the human race are also intimately intertwined. Human rights law translate morality and ethics into a rule, and provides their development. We can say that values, morality, ethics, law and human rights are all linked in a complex normative cluster, and the building of ethical and sustainable form of globalization is not exclusively a human rights matter, but it must include the recognition of shared responsibility for the universal protection of the human rights [13,14].

## The European Higher Education Convergence Process

Although we are now addressing the firm possibility of a common European Higher Education Area (EHEA), the background dates back to the publication of the Magna Charta Universitatum [15], signed in Bologna on 18 September 1988 on the occasion of the ninth centenary of its university. Its principles, which were clear and remain valid, were based on an awareness that the future of mankind largely depends on the cultural, scientific and technical base generated at universities, which undertake the task of disseminating knowledge to new generations and to society as a whole in the context of its cultural, social and economic future.

From this standpoint, the almost forced implication is that the University, in societies which are organized in a variety of ways depending on their particular geographical and historical characteristics, is an independent institution that passes on culture through research and teaching with a critical spirit, but ―and this is fundamental― with a moral and scientific independence alien to all political and economic powers. Therefore, freedom of research, teaching and training, as fundamental requirements, are the driving principle of the University. Through these principles, far from intolerance, the university becomes an exceptional place for encounters between faculty members, who have the capability of passing on knowledge through research and innovation, and students, who have the right, the will and the chance to be enriched by the process.

## European Convergence in Knowledge

The fundamental basis of the EHEA was laid down with the Magna Charta Universitatum. Ever since, European convergence in the field of higher education has been an ongoing process. Thus, the Sorbonne and Bologna Declarations of 1998 and 1999, respectively, marked the start of the process of convergence between the different national education systems for the implementation of a European Higher Education Area [16] by 2010. The Prague Communiqué (2001), signed by thirty-two countries, restated this aim, reflecting the conclusions of the meeting organized by the 2001 Conference of Rectors of Spanish Universities (CRUE) in Salamanca, the Convention of European Students held in Gothenburg the same year, and the activities of the European University Association.

The essential aspects of reform are associated with an education viewed from the perspective of learning; a structure and concept of degrees according to occupational profiles; a painstaking reflection on aims, skills and knowledge; the adoption of similar methodologies and the importance of updating subject-matter. All this involves a conceptual overhaul of the education systems to bring them into line with new training models focused on work and learning with the active participation of lecturers and students, reappraisal of contents, more personalized attention, and improved faculty coordination. In this way, the curriculum, considered as the theory and practice of planning and the process and evaluation of experiences in learning and teaching, should be organized in order to define a series of goals that may be translated, at the end of the learning process, in a set of student skills useful for his or her future career.

The curriculum shall specify what the student has to learn as well as offer guidance on future socialization in his/her professional field, transmitting not only scientific but also social and humanistic culture through promoting certain concerns and commitments. In short, the intention should be to streamline the learning and grading processes by means of the ECTS so as to assure student mobility within the European Union. The European Credit, as a unit of educational measurement, should assess the work done by the student in order to fulfil the objectives of the study schedule that will open the door for him/her to the European job market.

## Bioethical Universality in Biomedicine Knowledge

According to UNESCO, this aim will only be fulfilled when the quality of education is based on a high faculty research and teaching capacity, as the excellence of higher university education depends on scientific and pedagogical updating.

Those engaged in university teaching sometimes ask themselves, in view of the lack of ethics of some professionals ―amongst whom we may include researchers― whether we should consider attempting to transmit, besides theoretical and practical knowledge, the ethical "sense" (social state in which the equities are observed and the law is paramount) needed to research and pass on that knowledge and make the University a space for ethical learning as well. It is complicated, however, to teach something that originates from within the individual, as apparently nobody has ever instilled ethics and morality in us and, bearing in mind the characteristics of the human being, there are no teaching or social rules for evaluating good and bad. We could set out from a basic, easily understandable idea, namely that scientific knowledge is to be found to an overwhelming extent in nature and that, therefore, its discovery is not subject to limitations, but that does not mean that, as a human being, the researcher has to be so too. The limit is set, precisely, by bioethics when we refer mainly to Biology and Biomedicine.

The immediate question to be posed, however, is what Bioethics is from a conceptual point of view. Bioethics is the study of life ethics and the ways to be balanced against evolving social change. The ethics in Bioscience is designed to interaction between science and technology and established bioethical discourse, and demands an increased understanding of biological systems, the responsible use of technology and curtailment of ethnocentric debates more in tune with new scientific insights [17]. If we look back over history, the oldest meaning of ethics was of that of "The place where one lived", but according to the Greeks, rather than a physical place, it was an "inner" place, which the person reserved for him/herself. For Aristotlean doctrine, ethics and politics coincided, whereas for Kant, ethics represented an exacerbated individualism that sought one's own perfection. In Kantian ethics the concept of "motive" is the most important factor in determining what is ethical. More specifically, Kant argued that a moral action is one that is performed out of a "sense of duty". For Kant, a moral action is not based upon feelings or pity, and is not based on the possibility of reward, instead the moral action that is based on a sense of "this is what I ought to do". Besides these historical notes representing the origin of the present concept of ethics, we may say that ethics is a way of responsibly acting and assuming the consequences of one's own conduct. For this reason, the references are, inevitably, the categories of good and bad.

The origin of Medical Ethics [18,19] is contemporary with Socrates (469-399 B.C). In fact, if we recall the Hippocratic Oath [20], from that period, the premises envisaged in it are connected with swearing to the gods and, although medicine was not regulated as a profession, the concept of ethics was already flourishing. The Hippocratic Oath unites the physician as a human and as a technician according to its three fundamental maxims: benefit for the patient, alleviating his/her needs due to illness; the physician's professionalism and dedication, beyond corruption and personal interest, and confidentiality, preserving the patient's data and privacy. Thus, ethics and therapies should necessarily go hand in hand. Furthermore, it is obvious that this Oath is based on the logical rationality of an act, as is the case of healing, in which the most precious thing for man, namely his health, is at stake, its validity and effectiveness should be enduring over time, at least in their basic and general aspects.

The change in the concept of Medical Ethics took place in the 20th century ―and still continues today in our 21st century― such that it has entailed a re-think in the attitudes of researchers, doctors, patients, pharmaceutical companies and public authorities, although they often do not travel along the same path. The Nuremberg Code of 1947 laid the first stone of Biomedical Ethics by requiring the voluntary consent of subjects and minimum scientific requirements. Later, the Declaration of Helsinki [21], adopted by the 18th World Medical Assembly in 1964 and amended and extended in Japan in 1975, Venice in 1983, Hong Kong in 1989, Somerset West in 1996 and Edinburgh in 2000, were to lay its foundations.

In spite of all these international standards and declarations of good intentions in respect of Biomedical Ethics, in the 21st century many researchers still perceive a certain regression or, at least, a "lack of progress" in the development of ethically suitable research protocols.

The question may seem trivial, as we all have the idea that Bioethics is ―or should be― a universal concept and that, therefore, we ought not to raise the specific issue of whether it is possible to achieve European convergence in the teaching of bioethical principles, but the truth of the matter is that, in spite of everything, we continue to question whether Bioethics can be universal or not. The first thing we have to define is the concept of Bioethics and reach a consensus on an international level in general and a European one in particular, which in principle ought to coincide.

As fundamental rights relating to human and animal rights are often disregarded, it is easy to raise the question of whether it would not be necessary to teach the concept of Bioethics in the University as a plural concept for unrestricted action. Because we should not overlook that fact that, as we are reminded by Craig Venter ―the driving force behind human genome sequencing― the control over our biological destiny lies increasingly in our own hands.

## How to Teach Bioethics?

Objectively, it may be asserted that we can teach our students the essential points of ethics both in research and in any other activity connected with Biology or Biomedicine. First of all, we should consider each person's individuality when it comes to undertaking research work. With all its risks, this gives leads to our right of freedom as an individual, which in principle nobody can deny us. Secondly, bearing in mind that in recent times research has focused decisively on the study of different pharmacological molecules on patients themselves, it is mandatory, in these cases, to abide by the rules of ethical guidance laid down in the various international reports, codes and declarations. It is self-evident that if the research work has no scientific validity, i.e. it does not generate valid knowledge, by definition that work will not be ethical. Yet we cannot overlook its social validity, either, as properly compiled and presented scientific knowledge is the heritage of the society in which it is generated, and not for the researcher's own use and personal benefit, as the former invests social resources for this purpose; if this were not the case, science would not be ethical either.

Just as the United Nations Universal Declaration of Human Rights of 1948 may be considered a basic code of ethics, it is necessary to establish a Universal Declaration of Bioethics that is acceptable all over the world. In this respect, UNESCO has approved the Universal Declaration on Bioethics and Human Rights [22] in order to define the universal rules in the matter of Bioethics. This may represent the starting point for proper pedagogical guidance for Bioethics at the University both at worldwide level in general and at European level in particular, as it takes into account the democratic ideal of dignity, equality and respect for people and rejects all dogma of inequality between races and persons. In this way, scientific and technological development may be channelled in accordance with social change for the benefit of future generations.

The nature of person is a concept for philosophers but not for biologists. In ordinary language, "person" is often used synonymously with "human being". If we accept this, we would sort out the nature of persons by sorting out the nature of human beings, and the latter task certainly is best conducted by a biologist. However, in ordinary language, we distinguish between "person" and "human being" [23].

## Global Education in Bioethics in the European Higher Education Area

Article 23 of this UNESCO Declaration underscores the importance of Bioethics education, training and information to gain a better understanding of the ethical implications of scientific and technological developments. It is a repetition to some extent of what was postulated in the Universal Declaration of 1997 where governments were asked to further education and training in Bioethics across the board.

In this context, therefore, it is of the utmost importance to emphasize the need for those learners who are going to be engaged professionally in the scientific, environmental or healthcare fields "to be educated in Bioethics", but this will also have to apply to society in general. A start is naturally made on this task at the University as its "learners" will be those responsible for disseminating their knowledge objectively to society itself, although ethical dilemmas may often be created in the professionals themselves, who then debase the information they offer society due to a lack of objectivity influenced by their own ideologies or pressures of some other kind.

The EHEA provides a splendid opportunity to "educate" from the bioethical standpoint and not only on the basis of providing knowledge. The training of professionals must be integral with regard to the acquisition of professional skills, but at the same time in respect of other aspects of their professional work as a person, especially when the technological advances of recent times have opened up new opportunities for an involvement in human life that had never before arisen in the history of mankind. The global skills to be attained by students in the future EHEA in matters of Bioethics include:

• Knowledge of threats to any life form, appealing to the responsibility of human beings for the protection of biodiversity and the biosphere.

• Acknowledgement of the priority of the person over the interest of Science and of society itself.

• Non-discrimination on account of physical or mental state, social situation, disease or genetic characteristics and non-stigmatization of an individual or of a group.

• Knowledge of the need for free, reasoned and explicit informed consents for any clinical or scientific research and for any treatment or diagnosis in human beings.

• Independence in the assessment of research projects or the making of any decision connected with bioethical precepts, taking into account the different socio-cultural, religious and philosophical currents.

In the future EHEA, as a "multidisciplinary subject", Bioethics will provoke reflection on the complex world of science and ethics as a whole, combining scientific knowledge ―which has to be brought up to date at an ever-increasing rate― and anthropological and ethical knowledge. As a future professional, the student will have to assimilate that, in its relentless advance and unrestrained development, science is responsible for answering questions that are sometimes simple but at the same of the utmost importance. He/she will also have to assimilate that Bioethics represents the compass that guides responsibility for human actions in the scientific and professional sphere.

## Bioethics as a Connecting Link between the Credibility of Science and Society

As the new discipline of our century, Bioethics will make a contribution to the stabilization of the physician-researcher-patient relationship by introducing new values into the practice of medicine and research. Emphasis will be laid on the distinction between ethics and medical science, reducing an unnecessary and unjustified paternalism of the physician towards the patient in this relation, in the actual patient's benefit at all times. There will be an increase in the autonomy of patients in their decision-making capacity and, above all, in the respect for the people who, on certain occasions, in real life "act" as patients without their wanting to.

The researcher is not an individual isolated in accordance only with his eagerness to discover knowledge and truth. On the contrary, he is in fact immersed in a society that changes over time and which has to know how to adapt, as times change and the social assessment and requirement objectives with regard to science may change. In general, however, society places the researcher in a central position, in the knowledge that when all is said and done it is the scientists who, through their research work, can offer a solution to the problems arising in it of an economic, social or public health nature.

The force that science exerts on society should be based primarily on its capacity for solving social problems, passing on scientific knowledge and, essentially, on its credibility, which will stem from the need for its impartiality and objectivity. Because, if science is not credible, it will have no impact on society, but the fact of the matter is also that science can only be made credible if it is good science. This means it has to be based on the fundamental principles of ethics and on the most "severe" self-criticism, which are features that clearly differentiate it from the concept of ideology.

Researchers should adhere to the recognised ethical practices and fundamental ethical principles appropriate to their discipline(s) as well as to ethical standards as documented in the different national, sectoral or institutional Codes of Ethics [24,25].

## Some Conclusions

European Convergence is not only going to entail economic harmonization between the Member States, but also a commitment to a "twinning" process between people, as the new European Higher Education Area will involve convergence in the integral training and teaching of people themselves. But it is going to represent a challenge too in their joint technological advance, as the new technologies applied to Biology and Biomedicine will require new standards for performing an ethically consequential task, especially--as is now a reality-- when an individual's genetic heritage may be at stake, due to its possible impact on coming generations or, more immediately, on his closest social and working environment.

The future will be a question of uniting and converging by means of a single acquisition and application of knowledge and, for instance, of professional environmental, healthcare or research activities, where Bioethics will have to go hand in hand with these activities and convert them into a means of enhancing and increasing the progress of mankind.

The new Medicine studies in the European Higher Education Area will have to be brought into line so as to assure to the Bioethics a more significant role. This will have to be combined with an essential adaptation of the Medical Code of Ethics for students in which they impose on themselves a series of bioethical standards for performing clinical practices so as to enhance the physician's human qualities, his attitudes, his performance and relationship with the patient and with quality hospital practice.

## Competing interests

The authors declare that they have no competing interests.

## Authors' contributions

All authors read and approved the final manuscript.
